# “Xenotransplantation challenges us as a society”: What well‐informed citizens think about xenotransplantation

**DOI:** 10.15252/embr.202050274

**Published:** 2020-08-11

**Authors:** Johannes Kögel, Georg Marckmann

**Affiliations:** ^1^ Institute of Ethics, History and Theory of Medicine Ludwig‐Maximilians‐University of Munich Munich Germany

**Keywords:** Regenerative Medicine, S&S: Economics & Business

## Abstract

A citizen's conference on xenotransplantation delivers a cautious ‘Yes, but…’ endorsement. It also shows how additional knowledge and debate shifted peoples’ opinion on this technology.

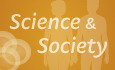

Xenotransplantation has come closer to clinical application. While it provides hope for patients with irreversible organ failure, it also raises ethical, psychosocial, and regulatory issues. These issues challenge us both as human beings and in our relationship to animals and should be discussed among broader society. In order to elicit well‐informed public opinion on the benefits and risks of xenotransplantation, we organized a citizens’ conference. After deliberating over three weekends, the participants drafted an evaluative statement on xenotransplantation and made recommendations to scientists and policy makers. Overall, the citizen group considers the benefits of xenotransplantation to outweigh the risks, but calls for strict regulatory measures to ensure the development of a sustainable and ethically justifiable biotechnology.

Overall, the citizen group considers the benefits of xenotransplantation to outweigh the risks, but calls for strict regulatory measures…

One hundred and ninety‐five days. This is the period of time denoted for a scientific breakthrough in the field of xenotransplantation (XT) (Längin *et al*, 2018). Baboons survived for 6 months with a genetically modified pig heart. Bringing xenotransplantation to patients with the prospect of reducing the current shortage of donor organs has thus become more realistic. This success owes to significant progress regarding the immunological challenges of XT on the molecular level and by better genome editing technologies such as CRISPR‐Cas9 (Sykes & Sachs, 2019). The time has come to align the necessary infrastructure of policy making, legal, and medical regulation, political administration, market interests, and public opinion.

While these advances in XT provide hope for patients with irreversible organ failure, they also raise ethical and philosophical issues: Is it ethically acceptable to breed animals and use them as an organic spare parts stock? What does it mean anthropologically for patients to live with an animal organ or animal tissue? How shall we balance the potential benefits and risks of XT therapies, given that everyone could potentially benefit as an organ recipient, but at the same time may risk acquiring a xenogeneic infectious disease? While the academic discussion on the ethical implications of XT has already started (Manesh *et al*, 2014), a broader societal debate is still lacking. This public deliberation may be more relevant for XT than for other bio‐medical innovations as it affects us as human beings and our relationship to animals. Therefore, the questions raised cannot be answered by reference to scientific evidence or ethical theories, but have to be deliberated in society. Not discussing these issues could result in an ethical rejection of promising XT therapies.

While the academic discussion on the ethical implications of XT has already started […], a broader societal debate is still lacking.

However, public deliberation about XT is especially difficult, as the topic is hardly recognized among the general population owing to its novelty and biomedical complexity. We therefore organized a citizens’ conference because it provides a useful tool to elicit well‐informed and carefully reflected opinions on the topic. The results of the conference, a written evaluative statement on xenotransplantation, the so‐called citizens’ vote, has been published online, debated publicly, and was sent to healthcare politicians. The goals of the citizens’ conference were to inform public debate and support policy and political decision‐making. The results seem especially relevant in that it marks the first public consultation since the aforementioned research breakthrough in XT, thus reflecting the latest stage of research and development. Public deliberation on XT has been considered especially necessary because “[t]o date […] ethical thinking has largely been left in the hands of scientists” (Jasanoff, 2018). For example, in Germany, there has hardly been any public debate on the current state of XT so far, nor has XT been considered by law or politics (Brown & Beynon‐Jones, 2010).

## “Yes, but…”—the citizens’ statement on xenotransplantation

Eighteen citizens, recruited through a multi‐stage process based on random and self‐selection, met over three weekends, during which they received information about XT, listened to and discussed with experts from various disciplines, and engaged in intensive group deliberations. At the end, they drafted a common statement on XT (Participants of the Citizen Conference on Xenotransplantation, 2019).

The right to kill animals for human purposes was discussed against the backdrop of a meat‐consuming society with vegetarians or vegans among the participants. The wide acceptance of meat production was, in itself, considered an insufficient argument for legitimizing XT. However, most participants held it to be justifiable to take an animal's life in order to save a human life. Nevertheless, a lot could and should be done to improve the animals’ breeding conditions.

The main argument for an overall positive assessment of XT was based on a societal obligation to help people with life‐threatening conditions and to alleviate suffering where possible. It was supported by the fact that even optimizing allotransplantation and the primary prevention of organ failure will not eliminate the shortage of donor organs. Therefore, it was considered unacceptable to infringe the individual's right to choose XT as a therapeutic option. This right requires that the respective individual has been empowered to make an autonomous decision based on comprehensive and balanced information. After all, it should be ensured that XT is implemented as a sustainable biotechnology. Notwithstanding, the citizens called for further improvements in the primary prevention of organ failures, as well as the promotion of allotransplantation and alternative approaches such as tissue engineering or stem cell research. In particular, the participants formulated several requirements for the acceptable use of XT (see Table 1).

**Table 1:** Participants’ requirements for acceptable development and use of Xenotransplantation

Research and development	Thorough investigations to assess risks of known and unknown diseases
	Provide comprehensive and balanced information to patients in XT trials
Ethical considerations	Equal access to transplants, free of any discrimination
	Medical urgency as central criterion for the allocation of allo‐ and xenotransplants, supervised by an independent organization under state control
	Thorough control of compliance with all animal welfare regulations; continuing research to optimize the conditions of animal husbandry
Psychosocial effects	Professional psychosocial care for XT patients
	Ensure a social climate free of stigmatization
Social responsibility	A balanced and sensitive approach to the topic of XT by the media
	Strict monitoring and control of XT regarding its development and implementation; guaranteed by parallel, interdisciplinary, and mutually controlling institutions
	Emergency measurements such as quarantine in the unlikely event of xenogeneic infections

## The effects of knowledge transfer and deliberation

**Figure 1:**
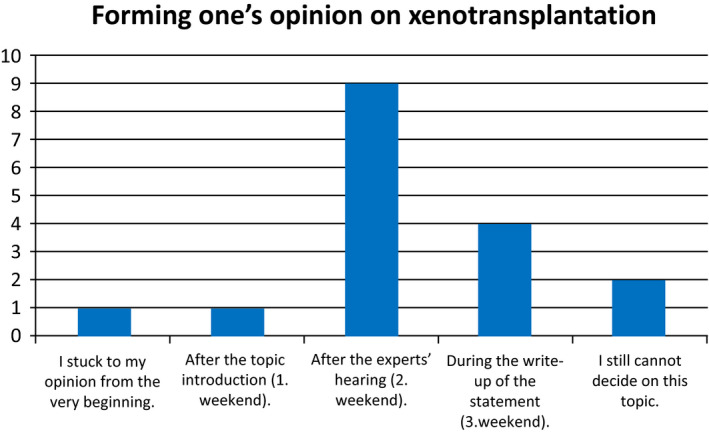
**Point of time when the participants of the citizens’ conference settled on their opinion regarding xenotransplantation**.

While most participants arrived with a rather critical view of XT, identifying more risks than opportunities, the majority finally endorsed an overall positive position, presupposing certain precautionary conditions. This change occurred after they acquired more facts and knowledge on XT, listening to the experts and discussing the topic among each other (Fig 1).

**Figure 2:**
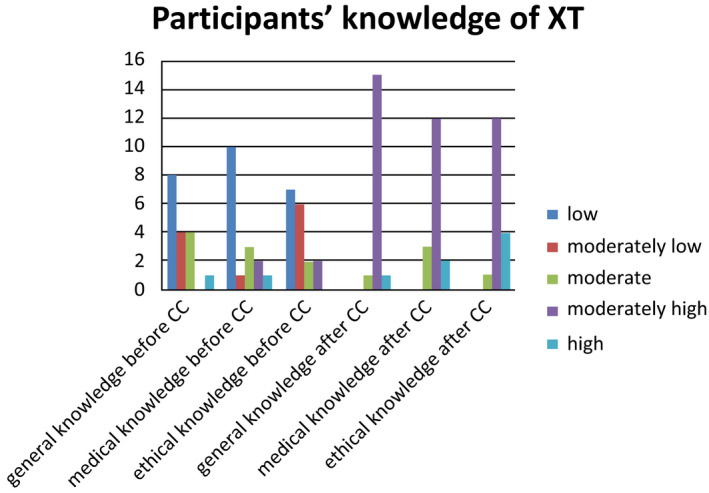
**Self‐reported knowledge of participants on the topic of xenotransplantation before and after the citizens’ conference (** **CC** **), assessed at the end of the conference (** ***N*** **= 17)**.

Two factors may have contributed to this change of opinion. First, the participants gained a lot of knowledge regarding XT in general and its medical and ethical implications in particular (Fig 2). Most participants changed their opinion after the second weekend when they received comprehensive information in the expert hearings. Relevant information may have included that a shortage of donor organs cannot be relieved completely by optimizing allotransplantation; that animals will be kept at least according to current regulatory standards; that a xenotransplant could be more beneficial for patients owing to its better quality and reduced need for immunosuppression; and that the risk for xenogeneic infections was considered extremely low according to latest virologic findings. Second, the deliberation of the citizens among each other in small groups and plenary meetings promoted transparency and self‐reflection of their arguments, points of view and attitudes underlying their individual opinions toward XT. The informal discussions during coffee or lunch breaks also influenced the opinion‐forming process. In the evaluation, the participants mentioned both aspects, gaining knowledge and group discussions including informal talks, as the key components in making up their minds about XT. Less important was, for example, the influence of the media, conversations with friends and family, or personal experiences related to the topic.

## “Xenotransplantation affects us all”

Even more remarkable than the change of opinion from a rather skeptical to a predominantly positive attitude was the participants’ sensitivity regarding the scope of XT. This is reflected in the statement that XT challenges us all as a society. This does not simply denote the fact that each of us may be affected by organ failure and in need of an organ transplant. It also considers the possibility of xenogeneic infectious diseases, which could spread among the whole population. Furthermore, it stresses the role of society as a whole in deciding about emerging biotechnologies and when citizens should have a say. XT affects and depends on fundamental tenets in our society. How shall we respond to illness and severe suffering and the resulting healthcare needs? Moreover, XT challenges the human–animal relationship in our society and raises the question to what extent we find it acceptable to instrumentalize animals for human purposes. Last but not least, the feasibility of XT will also depend on whether society responds to the recipients in a non‐stigmatizing manner.

## Conclusions

The citizens’ conference on XT demonstrates that citizen participation is not only possible, but that citizens are also willing and interested to get involved in topics of high societal relevance and are able to arrive at a well‐informed and carefully reasoned evaluation of novel and complex biotechnologies. With its balance of comprehensive information, exchange of opinions and collective deliberation, the citizens’ conference has proven to be indispensable in getting citizens involved and stimulating public debate (Blacksher *et al*, 2012). It also shows how important information is for developing a valid opinion regarding biomedical innovations. In an opinion poll, many participants probably would have rejected XT based on their originally skeptical position, while they arrived at a predominantly positive assessment after the expert hearings and joint deliberations. Insufficiently informed public opinions therefore could threaten important biomedical advances.

Regarding XT, the citizens clearly see and appreciate its potential to save severely sick patients’ lives. At the same time, scientists and policy makers must maintain citizens’ trust by ensuring transparency, rigor, and neutrality of regulation and controlling institutions. The various recommendations formulated in the statement may serve as a policy brief to political, legal, and scientific decision‐makers: Xenotransplantation yes, but …
